# The experience of young carers in Australia: a qualitative systematic review and meta-ethnographic synthesis

**DOI:** 10.1080/00049530.2024.2372266

**Published:** 2024-06-27

**Authors:** Owen Walker, Richard Moulding, Jonathan Mason

**Affiliations:** Faculty of Psychology, Counselling & Psychotherapy, The Cairnmillar Institute, Melbourne, Australia

**Keywords:** Young carer, experience, qualitative systematic review

## Abstract

**Background:**

Many people require additional care and support to meet their personal, health and psychosocial needs. Sometimes that responsibility falls to young people within their families. The research to date indicates that the impact on these young people can be significant, however no comprehensive reviews have yet examined this in Australian participants.

**Objective:**

Given the personal, subjective experience of caring, this study systematically reviews the qualitative literature on Australian youth under 25 who provide an informal caring role, using a meta-ethnographic approach.

**Method:**

Qualitative studies published since 2002 using Australian participants were included. The databases used included: Psychology and Behavioural Sciences Collection, PsycInfo, PsycArticles, MEDLINE Complete, Academic Search Complete, CINAHL, and Child Development & Adolescent Studies. Quality ratings and risk of bias are discussed.

**Results:**

Seventeen studies were included in the final analysis, representing the experiences of 531 young carers. The results suggest that the young carer experience is profound and impacts a range of domains, including social, academic, and psychological. The impacts are largely perceived as disadvantageous, although some positive outcomes are discussed.

**Conclusion:**

On the basis of the findings, the review concludes by offering a model to conceptualise the Australian young carer phenomenon.

## KEY POINTS

**What is already known about this topic**:

(1) Young carers perform a wide variety of important social, personal and healthcare tasks for family members.
(2) Being a young carer impacts physical and mental health, as well as academic and vocational prospects.
(3) Structural health and social care differences make international comparisons of the young carer experience difficult to undertake reliably.

**What this topic adds**:

(1) Systematically collected and synthesised qualitative information on Australian young carers is presented for the first time.
(2) Key themes relating to how they became young carers, their disconnection from support services and the psychosocial toll of undertaking the caring role emerged as important aspects of the young carer experience.
(3) Positive aspects of the young carer experience also emerged, but these require further study, alongside factors that mediate the impact of negative health and psychosocial outcomes.

## Introduction

Young carers exist across the world, undertaking a variety of roles for a range of family members (Becker, 2007). While the “caring” literature is relatively extensive, significantly less attention has been paid to adult carers’ younger counterparts. A review of the literature indicates that a caring role often falls to young family members, and the impact of caring at a young age can be extreme; young carers are often constrained to the home for many hours each week, severely impacting academic and social engagements (Lacey et al., 2022), often resulting in academic disadvantage, social isolation, and the development of anxiety and depression (Siskowski, 2006). However, “young carer” is an umbrella term that captures many different relationships: young carers provide care for different family members, at different life stages, with different care needs. Caring tasks include dispensing medication, providing emotional support, and assisting with personal hygiene (Saragosa et al., 2022). According to Carers Australia, there are approximately 235,000 young carers in Australia, and similar statistics are found in the United States and United Kingdom (Aldridge, 2018; Hendricks et al., 2021). There is a scarcity of research on young carers outside of high-income predominantly English-speaking countries and thus prevalence statistics are largely unknown. As such, it is a difficult population to research; the nuances of the caring role, as well as how these may influence support needs and outcomes, are not well understood. Less still is known about the way in which the consequences of being a young carer impact an individual’s transition into adulthood and beyond.

Previous literature also tends to generalise impacts of the caring experience across high-income predominantly English-speaking nations; however, little evidence exists to support this narrative when it comes to young carers. While it may be possible, given that there is significant cultural overlap between nations, this needs to be investigated given it is clear that there are vastly different approaches and levels of understanding when it comes to young carers (Becker, 2007). As stated by Becker (2007), the policy and service delivery responses vary considerably across the UK, USA, and Australia, which are three main global sources of young carer literature. Additionally, many high-income predominantly English-speaking nations have different academic, social, healthcare systems (Farmer, 2015; Wendt et al., 2012), and differences in attitudes towards multiculturalism (Metz et al., 2016). A comparison across 6 European nations found that legislation, frameworks, and policies related to young carers varied broadly (Leu et al., 2022), and another study comparing international attitudes towards young carers reports that, while young carers look “similar” across high-income nations, there are “country-specific nuances, variations, and differences” (Leu & Becker, 2017, p. 3).

The systematic reviews undertaken to date tend to include only a small number of Australian studies (e.g., Alfonzo et al., 2022; Lacey et al., 2022). In Alfonzo et al. (2022) systematic review, the two included Australian studies found inconsistent results, with one indicating that young carers experienced poorer mental health outcomes the more significant the caring role (King et al., 2021), while the other (Pakenham et al., 2006) found that young carers and their peers did not differ on mental health outcomes. The systematic review undertaken by Lacey et al. (2022) included only one Australian study; that written by King et al. (2021). Several reasons for the lack of Australian studies in global reviews have been proposed in the literature, including the poor quality of many Australian-based studies, and a preference for studies using quantitative methodology. While this may be the case, the inclusion of so few Australian studies does little for Australian society’s understanding of the phenomenon. Despite a modest breadth of published research around the world, the vast majority is undertaken using samples from high-income predominantly English-speaking nations, and there is minimal research exploring the nuances between countries. Any differences in national awareness, welfare initiatives, and cultural expectations of childhood development on the young carer experience remain unknown. There are also major factors that impede the accuracy of cross-nation comparisons. For example, young carers are referred to as a “hidden population” because evidence suggests reluctance to self-identify (Smyth, Blaxland, et al., 2011). Additionally, the definition of “young carers” varies widely, even within countries. For example, some institutions have an upper threshold of 18 (e.g., Centrelink, the Australian government welfare department), while others extend to 25 (e.g., not-for-profits such as Little Dreamers Australia). Given significant variation in the categorisation of young carers, and the lack of depth of research, a broad approach is warranted when exploring the literature.

As such, a comprehensive analysis is warranted which includes a broader range of publications, while still making reasonable attempts to minimize any possible negative impact on the quality of the synthesis. Additionally, much of the literature on young carers is qualitative in design. Qualitative research is well suited to exploring abstract and poorly understood phenomena. Unfortunately, such studies are often less likely to be included in a general systematic review (Graebner et al., 2012; Seers, 2015), and this is indeed the case when it comes to the young carer literature. Even among global systematic reviews that exclusively examine qualitative data, often very few Australian studies are included. This may be a reflection of the small scale, and perhaps overall quality, of Australian literature on the topic. Alternatively, it is possible that this represents a limitation of the existing systematic literature reviews. For example, Saragosa et al. (2022) conducted a recent qualitative systematic review to synthesise global young carer data and identify how they interact with health services and only one Australian study was included. Similarly, Rose and Cohen (2010) conducted their own systematic review exploring young carers’ accounts of the caring role and also included only one Australian study, albeit a different study than included in the Saragosa et al. (2022) review.

Given the above description of the current research landscape, it is clear that a systematic review that exclusively explores the Australian young carer phenomenon is warranted. Research in this field has proven to be difficult; young carers are a heterogeneous population, and it is hard to generalise findings across various subgroups. Additionally, young carers are often reluctant to self-identify, raising issues of sample and selection biases. Lastly, quantifying and conceptualising “care”, for example, the differences and similarities between young carers and children or siblings of someone with care needs is a difficult task. In combination, these difficulties are appropriately addressed by focussing on the qualitative literature.

Whilst previous researchers have used qualitative findings to generate a conceptual model of the young carer experience (Saragosa et al., 2022), the data used to inform this model includes only one Australian study. The aim of this systematic literature review is to accurately capture the current Australian young carer qualitative literature, and to synthesise the findings using a meta-ethnographic approach. A meta-ethnographic design allows for all findings to be compiled, interpreted, and then restructured into an overarching theory or conceptual model (Britten et al., 2002), ultimately permitting the development of a model specific to the Australian young carer experience that may be used as a conceptual platform to direct future research.

## Method

### Design

The review followed PRISMA guidelines (Page et al., 2021) and involved three general steps: systematic searching of the literature, generalised critical appraisal of the literature, and a narrative synthesis. Given that only one contemporary explanatory model exists for young carers worldwide, and no current model exists which specifically examines the young carer experience in Australia, a meta-ethnographic approach was selected as the framework for the narrative synthesis (Britten et al., 2002). Meta-ethnography is a commonly utilised research method for synthesising qualitative data (Sattar et al., 2021). This method not only requires the collection and synthesis of the data in order to cross-compare, but allows researchers to develop overarching themes to formulate a broader, original model which aims to explain the phenomenon in a practical and meaningful way (Noblit et al., 1988). This study formed part of a doctorate-level thesis and was ineligible to be pre-registered with PROSPERO.

### Search strategy and data collection

A literature search was conducted using EBSCOhost and included the following databases: Psychology and Behavioral Sciences Collection, PsycInfo, PsycArticles, MEDLINE Complete, Academic Search Complete, CINAHL, and Child Development & Adolescent Studies. In addition, a manual search through Google Scholar was also conducted. The search was conducted in February of 2024, and included studies published from 2002. This date range was set to ensure the included studies were largely contemporary and relevant, and given that the first major Australian article describing the young carer phenomenon was published in 2002 by the Australian Government. The search terms were: (Young OR Youth OR Young Adult OR Adolescent OR Family OR Kinship, AND car*), OR (Parent OR Sibling, AND Mental illness OR Disability OR Substance OR chronic), OR (Family care*), OR (Parentif*), OR (Adultif*) (see supplementary materials for the full search grid). Reference lists of the studies included at the abstract screen were manually examined as well.

**Figure 1.**
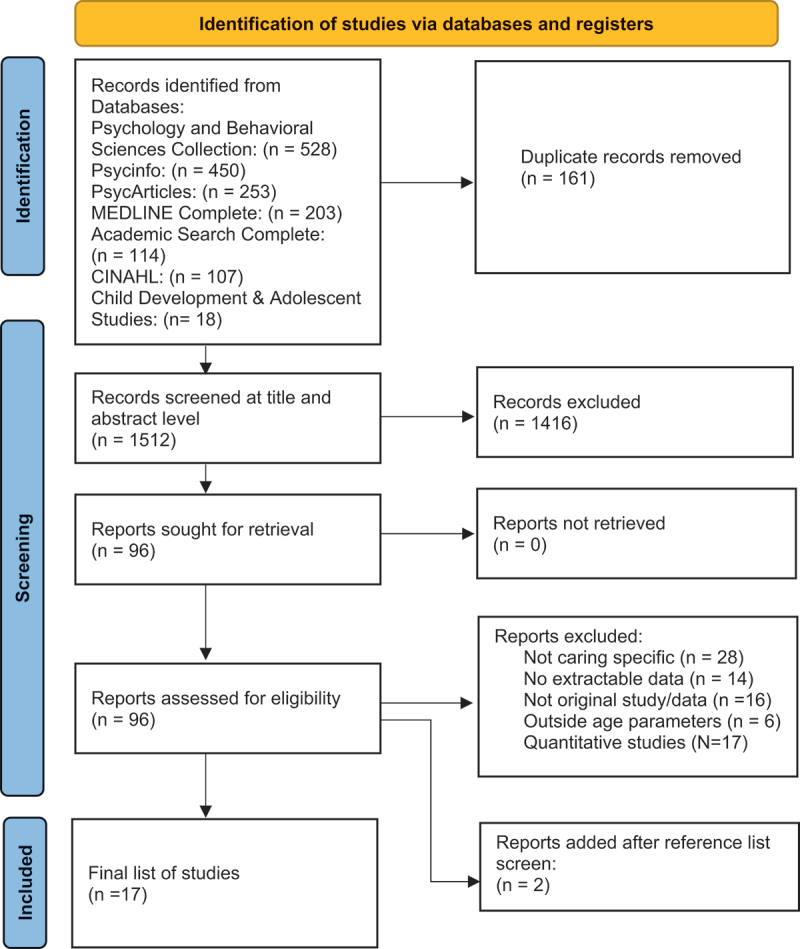
Flowchart of systematic literature review process (Page et al., 2021).

The included studies were then screened by the first author based on title and abstract. Of the remaining studies, 20% were screened by two co-authors (10% per collaborator; JM and RM). Once agreement was reached the remaining studies were screened at the full text level by the first author. Figure 1 depicts the literature review process, following the PRISMA guidelines (Page et al., 2021).

### Selection criteria

The following inclusion criteria was used:

- Original qualitative studies which used original datasets, or mixed-methods studies provided the qualitative data were reported separately in sufficient detail to extract, and;
- Included participants who undertook an informal (i.e., unpaid, non-professional) caring role for a family member prior to the age of 25, or where data for carers under 25 was reported separately and in sufficient detail to extract, and;
- Written in English, and;
- Using an Australian sample, and;
- Published from 2002-2024.

Studies were excluded if:

- There was merely a care recipient within the family (i.e., child of a parent with a mental illness) and no explicit mention of the caring role undertaken by the participants

### Critical appraisal

The academic and scientific rigour of each study was appraised using the JBI Checklist for Qualitative Research (Lockwood et al., 2017). The Checklist consists of 10 items that probe the methodological ethics and integrity of the research. The authors provide no standardised scoring methodological nor and categorical qualifiers to accompany the ratings. Risk of bias was assessed using the ROBINS-I tool (Sterne et al., 2016). This tool prompts researchers to consider the evidence for a range of potential biases. The number of criteria met for the checklist is included in Table 1 and an overview of the study characteristics is provided in the results section. No study was excluded based on quality in the interest of undertaking a realistic synthesis of all available literature.

**Table 1.** Characteristics of included studies.

Study	Year	Authors	Data collection method	Sample size	Age range	Gender ratio (M,F)	Ethnicity	Details of care provided	Checklist items met
1	2018	McDougall et al.	Semi-structured interviews	13	14–25	6,7	Aus, UK	12 = current YC, 1 = former. 4 = primary carers, 9 = secondary.	9
2	2011	Smyth et al.	Focus groups, interviews, and a short survey	68	12–25	NA	NA	Physical (ADLs), household (ADLs), medical, emotional, social support	10
3	2009	Moore et al.	Structured telephone interviews and a group interview	51 (44 phone, 7 group interview)	12–21	22,29	Generally non-Indigenous nor culturally or linguistically diverse.	21 primary carers, 30 secondary. Relatives with physical, intellectual, sensory disabilities.	7
4	2022	Dawes et al.	Semi-structured interviews	16 children, 10 adults	6–10, 4–18	13,13	Asian (31%), European (8%), Australasian (61%)	Family member with ABI	9
5	2017	Hamilton and Cass	Semi-structured interviews	30	17–25	NA	NA	Personal, household, emotional care.	6
6	2010	Moore et al.	Semi-structured interviews and activities	15: 12 interviews 3 in a focus group)	11–17	7,8	NA	AOD	9
7	2010	Smyth et al.	11 focus groups across interviews, and a short survey.	68 young carers, 16 service providers	11–25 (young carers)	NA	NA	NA	9
8	2007	Moore and McArthur	Face to face, semi-structured interviews	50	9–24	26,24	9 First Nations, 10 CALD	Care for family member (AOD, MI, disability).	8
9	2010	Moore et al.	1:1 interviews and a workshop	15	11–17	7,8	NA		8
10	2012	Hamilton and Adamson	Semi-structured interviews	36	7–25	19,14	NA	Family members with (AOD, MI, disability)	8
11	2021	Day	Semi-structured interviews	13	18–25	1,12	NA	62% cared for parent, 31% sibling, wide range of care needs.	10
12a	2005	Morrow	Questionnaires	54	6–18	31,25	Aus, India, NZ, Singapore	Physical, intellectual, psychological, Other.	6
12b	2005	Morrow	Semi-structured interviews with members of community	Unclear	NA	NA	NA		6
13	2004	Fudge and Mason	Focus groups, peer interviews	58	7–20	22,28 (8 NA)	NA	Parent with a mental illness	8
14	2010	Foster	Unstructured narrative interview and written narrative data.	10	25–57	2,8	Primarily Anglo-Saxon and Australian	Parents with mental illness: 9 mothers, 2 fathers	8
15	2011	Reupert et al.	Semi-structured interviews	12	8–15	6,6	1 indigenous	Mother/father, dual diagnosis	8
16	2018	Watson and Fox	Interviews using participatory methodology	12	12–17	6,6	NA	primary carer for a family member with physical or mental health problem	10
17	2021	Dunkley-Smith et al.	Semi-structured interviews	10	18–25	3,6,1 NB	White/European	Parents with MI	9

All participants were young carers unless specified. YC refers to young carers (typically 6–25 years old. The quality of each study was assessed using the 10-item JBI Critical Appraisal Checklist for Qualitative Research (Lockwood et al., 2017). NA means Not Available, indicating that this information was not available within the publication. ABI refers to acquired brain injury. ADLs refers to Activities of Daily Living. AOD refers to alcohol and other drug disorders. MI refers to mental illness. BPAD refers to Bi-polar affective disorder. NB refers to nonbinary.

### Data extraction and synthesis

The following study characteristic were extracted: title, year, author, design, method, study inclusion/exclusion criteria, aim and author conclusions, limitations and future research suggestions. The following sample characteristics were also collected: sample size, gender ratio, age range, race/ethnicity, care type/recipient (Table 1).

First and second order data and findings were imputed into Nvivo 14 (released in March 2020) for ease of interpretation. First order data refers to the participants’ responses, while second order data refers to the researchers’ interpretation of the data (Cahill et al., 2018). In line with the meta-ethnographic approach, the findings were read through multiple times by the first author, and cross-checked with the second and third author, to draw cross-study links between appropriately matched themes, and to understand where any cross-study conflicts arise. Both confirmatory themes and conflicting themes are then grouped using superordinate themes. The superordinate themes represent the *third order* information, i.e., the overarching cross-literature interpretations. These were elicited through back-and-forth discussion between the research team. The results were used to develop a conceptual model, utilising a meta-ethnographic approach.

### Cultural and theoretical framework

Theoretically, this study has been approached from a largely social constructionist, epistemological basis; a framework well-suited to qualitative analysis, particularly in the field of social sciences. Constructivism rejects the notion of an objective reality, and instead places emphasis on the importance of subjective experience and the narrative of the individual (Mills et al., 2006). Due to the narrative nature of any qualitative systematic review, including a meta-ethnographic approach, the relationship between researcher and objectivity is essentially asymptotic: one can strive to reach it but it is never truly attainable. As such, it is important to note the context with which the researcher has approached this study for the reader to have appropriate context behind the findings. The first author was a Clinical Psychology Registrar, completing a Doctorate level thesis for a post-graduate Clinical Psychology degree. The first author is Australian, of Irish descent. The first author grew up in a young caring role and is motivated to research the topic due largely to personal experience. The second author was raised and trained in the UK before moving to Australia to work in the academic field of psychology. The third author was born in the UK but trained in Australia. Both the second and third authors are of Anglo-Saxon descent and neither have personal experience of being a young carer. Any possible bias in the study inclusion process was addressed by cross-verification with two separate supervisors. Any bias in the thematic interpretation has been addressed in the same way.

## Results

### Study selection

A total of 1673 articles were returned from the initial search (see Figure 1). Title and abstract screening were completed by the first author. Of the remaining studies, 20% were screened by two co-authors (10% per collaborator; JM and RM). Once agreement was reached the remaining studies were screened at the full text level by the first author.

Seventeen articles were included in the final report, one of which contained two independent studies that were discussed individually. The vast majority of studies were exclusively qualitative, with a very small number including mixed data. Most studies relied on semi-structured interviews, while five also collected data through workshops and focus groups.

The oldest study that was included was published in 2004 with the most recent study published in 2022. The 17 studies were approximately evenly distributed across the date range. There was also a reasonable breadth of authorship, with the most prevalent author appearing across 4 of the 17 studies. All included studies used Australian samples.

### Study characteristics

**Figure 2.**
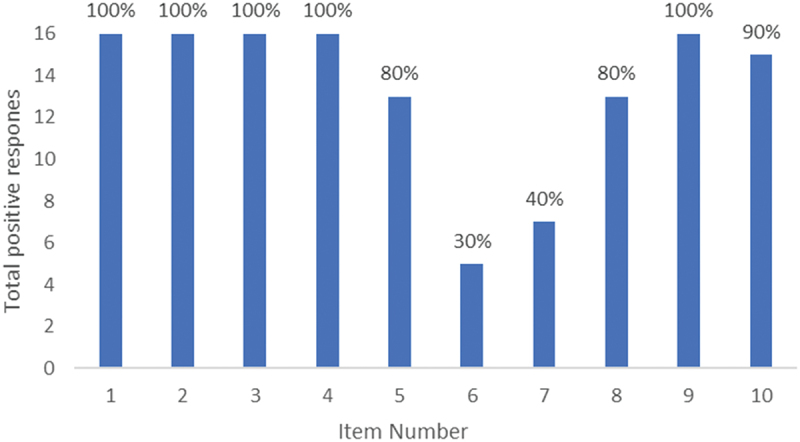
JBI checklist aggregate responses.

Most studies explored the caring phenomenon as it was occurring, as told by the young carer themselves, although there was one study which explored the experience retrospectively, and a small number which included perspectives of non-young-carers about young carers. All studies met at least 60% of the JBI checklist items, with the vast majority meeting 70–90% (see Table 1). There were two items of the JBI checklist that were consistently missed; item six and seven (see Figure 2). Item six assesses whether the researchers’ have indicated their cultural and theoretical framework, while item seven asks if the bidirectional influence between the researcher and the research has been acknowledged. Sample sizes ranged from 10 up to 68, and almost all of the included studies relied on semi-structured interviews for data collection.

The maximum response rate was 16 per item as Morrow’s (2005, 2005b) included studies were scored as a single article.

### Critical appraisal of included studies

There are currently no guidelines for interpreting JBI critical appraisal tool scores (Munn et al., 2020). However, given that such a large number of studies omitted relevant information about the research team and their impact on the research, as shown in Figure 2, it is clear that important context for data interpretation is missing. Additionally, key participant characteristics were also frequently omitted, particularly with regards to the culture and ethnicity of participants. Given the importance of culture and ethnicity on family roles, expectations, and dynamics it is difficult to draw robust conclusions about the cultural implications of the reported findings, particularly so given the frequent omission of the researchers’ own cultural and theoretical frameworks. While qualitative studies typically have smaller sample sizes than quantitative studies, eight of the included studies had 15 or fewer participants (and one study which did not clearly report the sample size). Given the diversity of participant experiences that are encompassed under the “young carer” term, the generalisability of each study across the broader young carer experience may be limited. Lastly, the included studies span a period of 17 years, a factor that must be considered when collecting data on a cohort whose experiences are likely to change across time.

### Research integrity information

Risk of bias was not individually analysed. Instead, an overview was conducted using the ROBINS-1 tool as a general guide (Sterne et al., 2016). As with all qualitative exploration, bias due to confounding is high, given that the data often relies exclusively on the participants’ subjective experiences and their personal beliefs. Selection bias is particularly relevant across the various studies, given that many participants self-selected. Additionally, the population is often thought of as “hidden”, and therefore it is entirely possible that the young carers that presented for the study are not representative of the wider population. It appears that there was a moderate bias towards selection of Australians of Anglo-Saxon descent compared to national data (approximately 30% of Australians are born overseas; Australian Bureau of Statistics, 2020). Demographic information of the participants was rarely indicated and often the gender of the participants was also unavailable.

As all thematic analysis requires some level of subjective interpretation from the researchers themselves, the reader must be aware of the inherent risk of bias in the selection of reported results (Ritchie et al., 2013).

### Participant characteristics

Unfortunately, many studies did not describe the race or ethnicity of participants. Those that did reported that the majority were Australian of Anglo-Saxon descent, however some did include Asian, Indian, First Nations, and culturally and linguistically diverse (CALD) participants. The age range across young carer participants was broad: the youngest participants were 6, and the oldest 25. One study, which examined the experience retrospectively, included adults up to 57. Two reports specifically explored the experience of young adult carers, and therefore restricted the participants to the range of 18–25. Gender split of all included participants was skewed modestly towards female participants (60/40), however four studies did not provide information on gender distribution. Many of the studies did not provide their explicit definition of a “young carer”, but most insinuated that they included young people up to age 25 who provided some level of care for a family member. Overall, the participants represented a wide variety of caring circumstances (physical, mental health, substance use, intellectual disability, etc.), and a diversity of care recipients.

### Synthesised findings

Grouping and cross-comparison of the first and second order constructs across all studies reveal four main third-order constructs: (1) the onset of the role, (2) the role itself, (3) supports, and (4) the psychosocial toll. Within each of the superordinate constructs are a range of subthemes, which are detailed in Table 2.

**Table 2.** Distribution of themes across the studies.

Theme	Authors																
	McDougall et al. (2018)	Smyth, Blaxland, et al. (2011)	Moore et al. (2009)	Dawes et al. (2022)	M. Hamilton and Cass (2017)	Moore, et al. (2010)	Smyth, Cass, et al. (2011)	Moore and McArthur (2007)	Moore, McArthur, et al. (2010)	M. G. Hamilton and Adamson (2012)	Day (2021)	Morrow (2005, 2005b)	Fudge and Mason (2004)	Foster (2010)	A. Reupert et al. (2011)	Watson and Fox (2018)	Dunkley-Smith et al. (2021)
**Onset**																	
Born into Caring	X				X					X							
Natural Progression					X					X				X			
**The Role Itself**																	
Diversity of Tasks	X	X	X	X	X	X		X	X	X	X	X	X	X	X	X	X
Diversity of Care Recipients	X	X	X	X	X	X		X	X	X	X	X	X	X	X	X	X
Uncertainty & Unpredictability	X		X		X	X			X		X	X	X	X	X	X	
Attitudes Towards Caring	X	X	X	X		X	X	X				X	X	X	X		
**Supports**																	
Lack of Access	X		X	X		X		X	X			X	X				X
Denial or Ignorance of Need	X							X				X	X	X			
Distrust	X		X			X		X	X					X			X
Respite	X	X	X			X			X			X	X				
**Psychosocial Toll**																	
Social	X		X		X				X			X	X	X			
Academic	X	X	X		X	X			X	X		X	X				
Psychological	X	X	X		X	X			X	X		X	X	X	X	X	X
Constraints	X				X	X				X	X			X			
Family & Relationships	X	X				X		X	X			X	X	X	X		
Benefits		X										X	X				X

### Onset

#### Born into caring

Almost all studies which explored the onset of the caring role described the role beginning naturally and from a very young age, with two studies both including quotes from participants who felt that they were “born into caring”, adding that they do not remember a specific onset period. The role began often through the normalised family experience, or a begrudging sense of duty and obligation.

#### Natural progression

In addition to the early onset of the role, participants across three studies reported that the role grew with them over time: as young carers became older, they were able to take on more responsibility and a wider range of roles. Likewise, the nature of the role would also change as the health and care needs of the recipient changed across time.

### The role itself

#### Diversity of tasks

Across almost all included studies, it was clear that the tasks required of young carers are diverse. These included physical care requirements, emotional support, domestic duties, healthcare responsibilities, practical support, and supporting other family members around the care recipient. Naturally, the role would vary depending on the care needs of the care recipient, but many of the participants would provide support across a range of domains.

#### Diversity of care recipients

Care recipients included parents, grandparents, and siblings. Sometimes young carers would act in a parental role for the siblings without formal care requirements, due to the incapacity of the parent (who may be the care recipient themselves, or who may be occupied undertaking the primary carer role).

#### Uncertainty & unpredictability

Uncertainty and unpredictability were common factors amongst many studies in one form or another. For many young carers, their caring role was unpredictable due to the nature of the care recipient’s fluctuating health. For others, the behaviour of the care recipient was unpredictable either due to behavioural disturbance from a disability such as autism, or unpredictability associated with substance use. All of these experiences were exacerbated by another relatively common factor in caring families: financial difficulties. This was due to a range of factors, including the care recipient’s treatment and support costs, and the frequent inability for the parents to work full time as they were either the care recipient or shared in the caring role. Families were often single parent families, which often meant the young carer was employed in a part-time job to support the family.

#### Attitudes towards caring

Many young carers reported feeling different to their peers and having a sense of “missing out” on their childhood due to the care requirements. Many believed that they were the only young carer in their social network, and this led to a sense of isolation and sometimes hatred towards the caring role. Despite this, many young carers reported feeling appreciative and grateful for the positive benefits of the role.

Many young carers chose to not identify themselves as such due to fear of bullying or intervention, or a lack of awareness that supports may be available for young carers. Young carers were often unlikely to view their caring tasks as unusual, as it was often normalised within the family.

### Supports

#### Lack of access

Many young carers across the studies report a belief that there are no services which can support them, although they were often aware of numerous supports for the care recipient. Alternatively, many young carers were unable to access supports due to not identifying as a young carer, or not being identified by those around them, including by their schools. Participants also commonly reported not having a trusted adult in their life who they believe would be able to guide them to any support services available.

#### Denial or ignorance of need

Often the young carer was prevented from accessing supports due to their own denial of the need, whether this was through normalisation of the role, or a minimisation of the impact they experienced. Often this was driven, or exacerbated, by the family not understanding, or denying, the impact on the young carer.

#### Distrust

Young carers often felt different to their peers, and at times, believed that any potential supports would not be in a position to help them, because they would not be able to understand the uniqueness of the young carer experience. They also had difficulty trusting their peers (often for fear of being bullied or judged) and thus would rarely confide in them or seek them out for support.

#### Respite

The most commonly discussed support that was provided or desired was respite from the caring role. Many young carers identified that the requirements of the role were relentless, and thus they were rarely able to take a break. Even when they were away from the role, such as when they were at school, they often experienced anxiety and worry about the care recipient and the home environment. Many young carers also discussed the importance of opportunities to be able to connect with peers who would understand what they have experienced, such as through camps and support groups.

### Psychosocial toll

#### Social

The barriers for social connection were numerous. Firstly, young carers often described themselves as different to their peers and therefore felt unable to connect with them meaningfully. This was often driven by a sense of increased maturity and independence compared to their peers. Additionally, they were often reluctant to engage with their peers for fear of being misunderstood, judged, and bullied for their differences. Due to the diverse requirements of the caring role, many young carers were also unable to engage in social activities after school and on weekends, as they were required at home. Caring families were often financially disadvantaged due to the caring role, and thus, young carers were often unable to attend school excursions and camps. At times, young carers’ social capacity was further restricted by their own need to work part time. Many young carers were able to engage with peers through support groups or camps, and all who did spoke highly of the benefits of social support.

#### Academic

The academic impact of the caring role was varied. For some, they experienced difficulty attending school due to conflicting obligations, although more young carers were unable to engage with extracurricular activities such as school camps or school sports, due in combination to a lack of finances and to the requirements of the caring role. This led to an impairment in the young person’s ability to engage with school more meaningfully. This was likely exacerbated by young carer reports that the school faculty were often unable to recognise and support their needs.

For most, the academic impact was largely seen through their inability to focus at school and complete homework tasks. This was often a consequence of anxiety felt at school, as they worried about the care recipient or the situation at home, or at times due to fatigue because of the caring demands. Many young carers reported a negative impact on their ability to sleep well.

#### Psychological

The psychological impact of the caring role was widespread. Many young carers reported anxiety and depression, as well as self-esteem issues. Often young carers would experience frustration and strong feelings of anger. Fatigue and emotional exhaustion were commonplace, and sometimes young carers would engage in maladaptive coping strategies, such as excessive alcohol consumption. Ubiquitous was the recognition that the role led to precocious development, which came with a range of positive and negative consequences.

#### Constraints

Some studies, which explored older participants, indicated that young adult carers are bound in their decision-making by the caring role. For example, choices around work, study, and moving out of home were all strongly impacted by their sense of duty, or fear of guilt should they become less available to support the family.

#### Family & relationships

Many young carers felt anger and resentment towards the care recipient. Conversely, many young carers felt that they grew closer to their family through the shared experience and many also recognised the importance of having good family supports. The ability to make and maintain social relationships into adulthood remained constrained for a lot of young adult carers, as their caring role often continued in some form into early adulthood.

#### Benefits

Despite a large focus on the negative aspect of caring, many young carers were able to identify some benefits. The most common amongst these were maturity and independence, although this was not without caveats such as the previously mentioned rift this created when trying to relate to peers. Many young carers identified a stronger sense of compassion for themselves and for others. One study identified that the caring role can motivate young carers to perform well in school and the workplace in an attempt to escape the role, or to avoid ending up like their parents.

### Conceptual model

**Figure 3.**
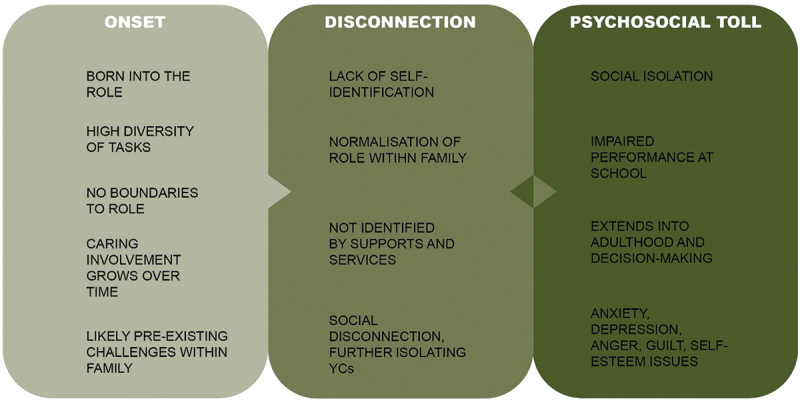
Visual representation of the young carer experience.

Figure 3 depicts a simple model visualising the young carer journey as described through the Australian literature. The model depicts three key points of difficulty for the young carer. Firstly, young carers (at least those represented in the literature) often find themselves in families already experiencing psychosocial difficulties. Young carers then grow into the role and through a range of factors are sealed off from supports, which further exacerbates the negative impact of the caring role.

## Discussion

The aim of this review was to synthesise and interpret the available literature to better understand the Australian young carer experience. The third-order constructs elicited through this review indicate that the young carer experience comprises numerous, inter-connected disadvantages, the effects of which compound over time and are often insurmountable.

Despite a significant range of adversities, some clear benefits emerge, such as maturity, independence, and compassion, although even positive traits were marred with negative connotations, as young carers often lament the divide these attributes create between themselves and their peers (Dunkley-Smith et al., 2021; Fudge & Mason, 2004; Smyth, Blaxland, et al., 2011). Analysis of the literature as a whole elicits a young carer journey, which commences as the young person assumes a caring role naturally but naively (M. G. Hamilton & Adamson, 2012; M. Hamilton & Cass, 2017; McDougall et al., 2018), driven by a normalised perspective of family roles and responsibility, or a sense of obligation to support the family (Foster, 2010; M. G. Hamilton & Adamson, 2012; M. Hamilton & Cass, 2017). They then experience significant adversity across a range of domains and numerous barriers to support, both internal and external to the family, which lead to the young carers feeling isolated, often (at least according to them) carrying the burden alone (e.g., McDougall et al., 2018; Moore, McArthur, et al., 2010). The only support many young carers receive is respite from the role itself, often involving connecting with other young carers (e.g., Smyth, Blaxland, et al., 2011). Most young carers, however, are not privy to such opportunities, and are rarely able to connect with other young carers. Young carers are also often disconnected socially from their non-caring peers, and therefore, are unable to learn from their peers about normative childhood roles and expectations (e.g., Moore et al., 2009). Because of this general social disconnection, in conjunction with a reluctance to disclose their caring circumstances, young carers are often oblivious to their proximity to other young carer peers (e.g., Foster, 2010; Moore, McArthur, et al., 2010).

This social disconnection happens during an important time in a young person’s development, where the typically developing adolescent begins to individuate (Blum, 2004). Social isolation for a young carer can also result in emotional isolation (and vice-versa). Alongside a paucity of other supports, it is expected that the burdens of the role may be internalised and thus significantly impair the young person’s capacity to engage in a healthy transition to adulthood. Essentially, young carers are insulated in an unpredictable and often demanding family environment with little reprieve and limited opportunities to develop the tools needed to appropriately navigate these challenges. There is a tension between wanting to escape the struggles of the role and being constrained by guilt and love for the family, which leads to significant mental health problems for young carers.

The young carer journey described above is depicted visually by Figure 3. The model presented in our research (Figure 3) emphasises the significance of the disconnection between the support needs of young carers and their access to support. Young carers are often unaware of available supports, such as government welfare initiatives such as the carer bursary program (Young Carers Network, 2024), or a range of psychosocial support programs run by non-government organisations (e.g.; Little Dreamers, 2024). Additionally, many young carers actively minimise or struggle to identify their own care needs. Likewise, the family unit itself is often reluctant to seek support, or unaware that supports exist, as the focus of families and services is typically on the individual with overt care needs. This phenomenon appears to be shared by young carers around the globe (Kavanaugh et al., 2016). While research is certainly required to develop sound interventions for young carers, their effectiveness would undoubtedly be improved by targeted research to understand how to best connect young carers to existing and future programs.

While the model begins to conceptualise a narrative of the young carer journey, there are significant gaps which warrant further exploration. As there is a paucity of research on the predisposing factors, it is unclear how representative the included young carer samples are of all families where a child undertakes a level of familial care. Likewise, the extent to which the impact follows the young carer into young adulthood and beyond is not well understood, as most of the literature focuses on the young carer experience told by the young person while they are actively in that role. Saragosa et al. (2022) also proposed a three-stage model to conceptualise the young carer experience, but their model demonstrated the chronology of the caregiving role in a linear fashion. Stage one comprised the initiation of the caregiving role, stage two comprised the act of caregiving, and stage three explored life post-caregiving. There are some similarities between the two models. Firstly, they found that the onset of the role is often gradual, and the responsibilities acquired are often burdensome. The two models also identified a significant emotional toll. The authors also report a significant physical burden, which was not found in the included Australian studies. This may be explained by differences in study populations, as a large number of Australian studies focused on care needs that were less physically burdensome (e.g., mental illness). Both models highlight the possibility for positive reflection and desirable outcomes such as maturity, but the global model includes elements regarding the young carer transition into adulthood and beyond, which is currently a gap in the Australian literature.

### Limitations

The purpose of this review was to provide a broad synthesis of Australian young carer qualitative literature. While the methodology served its purpose, and will hopefully provide a basis for further research, a significant limitation of this design is that it necessitates inclusion of an extremely heterogenous population. Additionally, the decision to examine the research across 22 years was also based on an anticipation of receiving a very small number of publications. Lastly, another contributing factor to the resulting heterogeneity is the broad age range. Many organisations define young carers as youth up to 25, but it is clear from this research that the experience is likely age-dependant. Given that 17 studies were included in this review, future research may be able to provide a narrower focus, for example by looking at particular age groups of particular care recipients, and therefore likely draw stronger conclusions. It is also worth noting that this review was not pre-registered with PROSPERO.

The majority of participants across all studies were Australians of Anglo-Saxon descent, therefore there was minimal discussion regarding potential cultural implications. Given the multicultural makeup of Australian society, understanding any influence of culture is imperative in order to understand the true Australian young carer experience (Boese & Phillips, 2011). Unfortunately, many studies included often lacked cultural and sociodemographic information of participants, and some studies did not provide any information on gender. Very few studies provided a robust theoretical perspective of the authors, nor did any place the author contextually within the research, which is important in any qualitative study as the author is inseparable from the research (Mitchell & Cody, 1993).

### Implications

It is clear from the findings of this review that young carers represent a vulnerable group of Australian youth. This research is a major step towards identifying the key areas of disadvantage, as well as eliciting key areas for potential intervention. While it is highly likely that financial and practical supports would alleviate much of the young carer disadvantage, further research would be required to understand the relationship between family resources and the young carer experience.

Many young carers report minimal awareness of their plight from others, and their own subsequent disconnection from any available services. It seems that increased awareness of the young carer phenomenon, and young carers supports could be beneficial. Further research is certainly required to understand the best way to provide such information to families.

Additionally, many young carers are able to draw some positive meaning from their role, and it appears that this process in itself is likely therapeutic. Young carers, therefore, may benefit from access to therapy, potential narrative style. Such an intervention could also be delivered in a group setting, which would also potentially address the shared experience of social isolation.

## Conclusion

The experience of caring for a loved one can be meaningful and empowering. However, when the role is thrust upon a young person, particularly one who is inadequately prepared and supported, the experience can be highly burdensome. According to the current literature, many young Australians unfortunately find themselves in such circumstances. They are subsequently impacted across academic, social, and psychological domains, and develop powerful emotional narratives about the negative aspects of their experience. Through a range of individual, familial, and societal factors, the young carer remains unacknowledged and unsupported, and the negative impacts compound across time, leading to detrimental outcomes. Thus, it is important that more is done to identify and support young carers, from both a social and a psychological perspective, to help them navigate the challenges of the role through childhood and adolescence, and to identify young carer needs as they transition into adulthood.
